# Interferon Signaling in Chickens Plays a Crucial Role in Inhibiting Influenza Replication in DF1 Cells

**DOI:** 10.3390/microorganisms10010133

**Published:** 2022-01-10

**Authors:** Daniel S. Layton, Kostlend Mara, Meiling Dai, Luis Fernando Malaver-Ortega, Tamara J. Gough, Kerri Bruce, Kristie A. Jenkins, Andrew G. D. Bean

**Affiliations:** 1 CSIRO Health and Biosecurity, Australian Centre for Disease Preparedness (ACDP), Geelong, VIC 3220, Australia; kostlend.mara@csiro.au (K.M.); meiling.dai@csiro.au (M.D.); tamara.gough@csiro.au (T.J.G.); Kerri.Bruce@csiro.au (K.B.); Kristie.Jenkins@csiro.au (K.A.J.); Andrew.Bean@csiro.au (A.G.D.B.); 2 Department of Biochemistry and Molecular Biology, Monash Biomedicine Discovery Institute, Clayton Campus, Monash University, Clayton, VIC 3800, Australia; luis.malaver-ortega@monash.edu

**Keywords:** IFNAR, interferons, innate immunity, interferon stimulated genes, antiviral response, influenza virus, viral infection

## Abstract

Influenza A viruses (IAV) pose a constant threat to human and poultry health. Of particular interest are the infections caused by highly pathogenic avian influenza (HPAI) viruses, such as H5N1, which cause significant production issues. In response to influenza infection, cells activate immune mechanisms that lead to increased interferon (IFN) production. To investigate how alterations in the interferon signaling pathway affect the cellular response to infection in the chicken, we used CRISPR/Cas9 to generate a chicken cell line that lacks a functional the type I interferon receptor (IFNAR1). We then assessed viral infections with the WSN strain of influenza. Cells lacking a functional IFNAR1 receptor showed reduced expression of the interferon stimulated genes (ISG) such as Protein Kinase R (*PKR*) and Myxovirus resistance (*Mx*) and were more susceptible to viral infection with WSN. We further investigated the role or IFNAR1 on low pathogenicity avian influenza (LPAI) strains (H7N9) and a HPAI strain (H5N1). Intriguingly, *Ifnar^−/−^* cells appeared more resistant than WT cells when infected with HPAI virus, potentially indicating a different interaction between H5N1 and the IFN signaling pathway. Our findings support that ChIFNAR1 is a key component of the chicken IFN signaling pathway and these data add contributions to the field of host-avian pathogen interaction and innate immunity in chickens.

## 1. Introduction

Avian influenza A (AI) virus, as a zoonotic agent, represents a significant threat to public health and poultry production worldwide. Outbreaks in farmed chickens is of great concern, as many AI virus strains, such as H5N1 and H7N9 can be directly transmitted to humans and in some instances lead to high morbidity and mortality. Furthermore, the impact of such virus strains with pandemic potential on human health would be devastating, given the lack of specific vaccines and the emergence of drug resistant IAVs. Infection with LPAI viruses in poultry often results in subclinical infections or causes mild respiratory disorder accompanied with reduced egg production and low mortality. Conversely, infections caused by HPAI viruses can induce an acute disease with fatality as high as 100% in domestic poultry. Additionally, AI outbreaks result in a significant cost to the poultry industry, as the only containment measures are the quarantine or mass slaughtering of the birds. It is therefore critical to further our understanding of LPAI and HPAI to ascertain risk and develop new preventative measures.

Innate immunity is the first line of protection against pathogen infection. A key component of innate antiviral immunity are IFNs, a class of cytokines with antiviral and immunomodulatory activity. Upon secretion, these molecules initiate signal transduction pathways leading to the up-regulation of the transcription of many genes, commonly named ISG, supporting an antiviral state in the surrounding cells [1]. In chickens, like mammals, there are three known classes of IFN, Type I, II and III. Type I IFNs were first identified by Isaacs and Lindeman [2] in influenza infected chicken embryos. Although these chicken IFNs have functional homologies with mammalian IFNs, chickens express a reduced numbers of Type I IFNs, in contrast to the different types of IFNs found in mammals [3]. Both IFNα and IFNβ bind to the chicken Type I IFN receptor complex (chIFNAR) which is comprised of two subunits, chIFNAR1 and chIFNAR2. Chicken IFNα and IFNβ share only 57% homology and the antiviral state induced by chIFNα has been shown to be stronger than that of chIFNβ, which is likely attributed to the greater expression levels of downstream antiviral ISGs [4]. In a study by Qu et al., the anti-VSV activity of chIFNα was 100-fold greater than the anti-VSV activity of chIFNβ, based on the cytopathic effect inhibition assay on DF1 cells [5]. The same study also demonstrated that a differential expression of ISGs, such as 2′,5′-OAS, PKR, IL-6, MHC-I, IFNAR-1 and IFNAR-2 was at the core of the different antiviral activities of both IFNs, with chIFNα showing a higher induction of these genes. Several studies, mainly in mouse models, have demonstrated that blocking the IFNAR receptor leads to high influenza viral loads, high mortality and decreased activation of ISGs such as PKR and Signal transducer and activator of transcription 1 (Stat1) [6]. Similarly, dendritic (DCs) and macrophages of *Ifnar^−^*^/*−*^ mice infected with Lymphocytic Choriomeningitis Virus (LCMV) showed higher levels of viral infection than wild type (WT) mice, as well as higher levels of viral nucleoprotein (NP) [7]. In PBS-12SF chicken cells, shRNA knock-down of IFNAR1 resulted in higher production of influenza H1N1 and decreased expression of ISGs suggesting a limitation in the antiviral mechanisms controlled by IFNs [8].

Upon ligand binding, the IFNAR receptors are activated and able to recruit further effector molecules belonging the JAK/STAT family, leading to the formation of the IFN-stimulated gene factor 3 (ISGF3) complex. This complex translocates into the nucleus, binds to IFN Stimulated Response Elements (ISRE) with consequential activation of the transcription of ISGs [9,10]. The ISGs play an important role in the antiviral response against the pathogen by acting at several stages of the virus replication cycle. Two of the most studied ISGs in chicken include the *Mx* and *PKR* genes [11,12]. Mx is a key antiviral protein involved in blocking the early stages of viral replication. In mammals, there are two forms of the Mx protein, MxA and MxB. Interestingly, only MxA has been reported to be a potent inhibitor of influenza [13,14]. In birds, there is just one lineage of the *Mx* gene [3]. Although the avian Mx protein shares structural homologies with the human protein, its antiviral activity requires further analysis, with studies reporting contradictory results. A number of studies have demonstrated that a variant of Mx containing an asparagine (Asn) at position 613 (Mx-Asn 613) has an antiviral effect against VSV and Newcastle Disease Virus (NDV) [15,16,17], whereas other in vivo studies were unable to clearly demonstrate an antiviral effect against H7N1, H5N1 and H7N7 [18,19], or showed only moderate reduction in mortality in chickens following H5N2 infection [20]. Another well studied ISG in chickens is *PKR* [21], which is activated by dsRNA and responses to Toll-like receptor (TLR) mediated immune responses. Upon activation, it phosphorylates eukaryotic initiation factor 2 (eif2) with consequent inhibition of mRNA translation in infected cells. In vitro studies on chicken DF1 cells have shown upregulation of chicken *PKR* following IFN stimulation [5] confirming the involvement of *PKR* in AI virus infection.

Whilst IFN signaling has been widely studied in mammals, it is clear that this pathway has yet to be fully elucidated in chickens. Thus, exploring the role of Type I IFN signaling may further elucidate the innate immune response involved in antiviral defense. In this study, we demonstrated that deletion of the Type I IFN response, by blocking the Type I transduction signal through deletion of the chIfnar1 gene, prevented activation by known ISGs. Furthermore, we showed that *chIFNAR1^−/−^* cells were more susceptible to virus infection, potentially due to a shorter virus cycle. Interestingly, decreased viral infection was found in *chIFNAR1^−^*^/*−*^ cells upon infection with HPAI H5N1 compared to WT cells, suggesting an important functional interaction with HPAI and IFN signaling. These findings may have implications for our understanding of the innate immunity in chickens and the antiviral response that is activated following influenza infections.

## 2. Materials and Methods

### 2.1. Cell Culture and Transfection Procedure

The chicken fibroblast cell line DF1 [22] (American Type Culture Collection number: CRL–12203) was provided by the tissue culture laboratory at the CSIRO Australian Centre for Disease Preparedness. The DF1 cells were maintained in Dulbecco’s Modified Eagle’s Medium supplemented with 10% fetal calf serum (FCS), 2 mM L-glutamine, 10 mM HEPES, 1.5% (*w*/*v*) *sodium bicarbonate*, 100  U/mL penicillin and 100  μg/mL streptomycin and incubated at 37 °C in a 5% CO_2_/95% air atmosphere.

### 2.2. Virus Strains

The Influenza virus strains used in this study LPAI Influenza A/WSN/33 (H1N1); HPAI Influenza A/Vietnam/1203/2004 (H5N1); Influenza A/Anhui/1/2013 (H7N9) were propagated by allantoic cavity inoculation of 9–11-days of embryogenesis specific-pathogen-free (SPF) embryonated chicken eggs. The virus stock was titrated in chicken eggs and the 50% egg infectious dose (EID50)/mL was calculated according to Reed and Muench [23]. All in vitro work involving Influenza A/WSN/33 (H1N1) was conducted within BSL-2 facilities at ACDP. All experiments with infectious HPAI Influenza A/Vietnam/1203/2004 (H5N1) and Influenza A/Anhui/1/2013 (H7N9) were conducted under BSL3 enhanced containment at ACDP approved by the CSIRO ACDP Institutional Biosafety Committee with Approval # PARA 2019/003.

Virus titrations were performed on Madin-Darby canine kidney (MDCK; ATCC #CCL-34) in flat bottom 96-well plates in DMEM supplemented with 0.5% bovine serum albumin and 1 μg/mL of TPCK trypsin. Cultures were carried out for 5 days and cytopathic effects were determined and TCID50 calculated.

### 2.3. Protein Modelling

The structure of chIFNAR1 was predicted by retrieving the amino acid sequence of both chicken (*G. gallus* AAU93528.1) and human (*H. sapiens* AAT49100.1) IFNAR1 from the Genbank database. The protein sequences were modelled using the Phyre2 webserver to create predicted protein structures for both the chicken and human IFNAR1 receptor [24].

### 2.4. CRISPR Guide Selection and Plasmid Construction

We used the RNA-guided Cas9 nuclease from the microbial clustered regularly interspaced short palindromic repeats (CRISPR/Cas9) system, to produce a dual double-strand break (DSB) by duplexing constructs encoding two guides RNA (sgRNA) as previously reported (Ran et al., 2013). Briefly, two sgRNA (GCCGCGTGCGCAGTCGTCAGAGG, left hand and AGCACCGGGACACCACGACCAGG, right hand); were cloned into to the pSpCas9(BB)-2A-GFP (Plasmid ID: 48138; ADDGENE) and transfected into the continuous chicken embryo fibroblast cell line (DF1). The two sgRNA acted together to produce a deletion in the chicken IFN (alpha, beta and omega) receptor 1, chIfnar1 gene (Gene ID: 395665). The expected deletion was 97 base pairs (bp). Cells Transfected by Lipofectamine™ 2000, were sorted using a BD FACS Aria II cell sorter based on their GFP expression. A second round of sorting was performed to obtain single clones for further expansion and genomic DNA (gDNA) PCR screening.

### 2.5. Genomic DNA Isolation and PCR Analysis of ChIfnar1 Gene

The identification of *chIfnar1* knockout (KO) cells was simplified to a quick gDNA PCR screening on clonogenic isolations of the cell lines after sorting. Genomic DNA from transfected and WT cells was extracted using DNeasy Blood & Tissue Kit (Qiagen, VIC, Australia) as per the manufacturer’s instruction. PCR was performed using GoTaq Flexi DNA Polymerase (Promega) following manufacturer’s instructions. Primers for screening were: Forward 5′-CGGCCACCCAAACCTTAGAA-3′ and reverse 5′-CCATCTCGCAGCAGTTGTCT-3′. to confirm the identity and extent of the deletion, amplicons were excised, purified from the gel (The Wizard^®®^ SV Gel and PCR Clean-Up System) and cloned into the pGEMt-Easy vector (Promega) for sequencing and analysis at Micromon Genomics sequencing facility (Monash University, Clayton, VIC, Australia).

### 2.6. Flow Cytometry and Cell Sorting

Influenza infected and uninfected cells were harvested and permeabilized with BD Fix/Perm solution (BD Biosciences, San Diego, CA, USA). The cells were then washed with Perm/Wash buffer and incubated with the primary antibody mouse anti-Influenza A Nucleoprotein antibody (Bio-Rad) for an hour at room temperature. The cells were again washed in Perm/Wash buffer and incubated with goat anti-mouse Alexa Fluor 488 (Life Technologies, Carlsbad, CA, USA) secondary antibody for an hour at room temperature. The cells were washed in Perm/Wash buffer, resuspended in FACS buffer (PBS, 4% FCS, 0.01% Sodium Azide) and analyzed using an LSR II flow cytometer (Becton-Dickinson, Franklin Lakes, NJ, USA). The data were processed using BD FACSDiva software (Becton-Dickinson, Franklin Lakes, NJ, USA) and analyzed with Flowlogic software (Version 7.2.1, Inivai Technologies, Mentone, VIC, Australia).

### 2.7. Quantitative Real Time PCR (qRT-PCR)

The RNA from DF1 cells was extracted using the RNeasy Plus kit (QIAGEN) followed by cDNA synthesis using the SuperSript III One-step RT-PCR system kit (Life Technologies, Carlsbad, CA, USA) according to manufacturer’s instructions. The relative quantitation of gene expression was determined using a Real-Time PCR System and the comparative threshold cycle (Ct) method was used to show change in gene expression according to manufacturer’s instructions (Applied Biosystems, Foster City, CA, USA). IFNAR1 transcription levels were analyzed using the Gg03338945_m1 assay (Cat# 4351372), primer/probe sequences were undisclosed. Relative gene expression was calculated using the mean values obtained from ΔΔCt relative to the endogenous control housekeeper gene glyceraldehyde-3-phosphate-dehydrogenase (GADPH). The qRT-PCR primers and probes (Table 1) used for the detection of chicken *IFNα*, *Mx* and *PKR* genes have been previously described [25,26].

### 2.8. Statistical Analyses

To determine the significant difference between uninfected and IFNα/IAV infected cells, a one-way ANOVA with multi-comparisons analysis was performed. Instead, to determine the significant difference between the percentage of infected vs. non-infected cells and the Mean Fluorescence Intensity (MFI) a Ratio paired *t*-test was performed. Alpha for all tests was set at 0.05 and results were considered significant if *p* values of less than 0.05 were obtained. Error bars represent the standard error of the mean (SEM).

## 3. Results

### 3.1. Characterization of the Chicken Ifnar1

**Figure 1:**
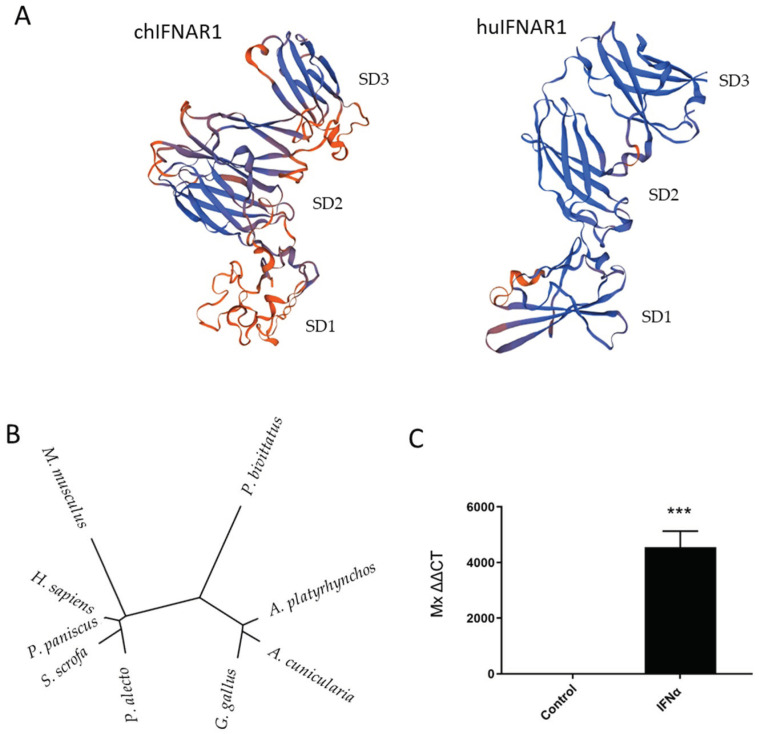
Chicken IFNAR1 is structurally and functionally similar to human. (**A**) Predicted tertiary structure of the ECD of chIFNAR1 compared to hIFNAR1. In blue are indicated β-sheets, in orange the coils. The four fibronectin type III (FNIII)-like subdomains are named SD1–SD4. (**B**) Phylogenetic relationship between IFNAR1 protein sequences from different species. The Accession Numbers of each IFNAR1 sequence were retrieved from the ensemble database. Chicken (*G. gallus* AAU93528.1), human (*H. sapiens* AAT49100.1), mouse (*M. musculus* AAH43935.1), duck (*A. platyrhynchos* XP_021124082.1), pig (*S. scrofa* CAJ76278.1), bat (*P. alecto* XP_006921038.1), snake (*P. bivittatus* XP_007442083.1), chimp (*P. paniscus* XP_003824035.1) and owl (*A. cunicularia* XP_026702394.1). (**C**) IFNα stimulates Mx through IFNAR1 on Chicken DF1 cells. Chicken DF1 cells were stimulated with ChIFNα. Cells were harvested for RNA isolation, and cDNA preparation. Expression of Mx in control (white) and IFNα (black) stimulated cells was assayed by real-time PCR. Data were shown as mean ± SEM (n = 3). A value of *p* < 0.05 was considered statistically significant. Data marked with (***) indicate *p* < 0.001.

While the structure of human IFNAR1 has already been characterized, little information is available regarding the structure and function of chIFNAR1. Structural analyses between chicken and human IFNAR1 receptor revealed structural homology between the two proteins. Similar to the human counterpart, chIFNAR1 structure is composed of three main structural domains, an ectodomain (ECD), a transmembrane and a cytosolic domain. This homology in structural architecture between chicken and human IFNAR1, suggests a similar function of the receptor. Furthermore, we evaluated the phylogenetic relationship between IFNAR1 amino acid sequences from multiple species. Based on the clustering patterns and sequence homologies, the phylogenetic analyses clustered the proteins in three groups. Although chIFNAR1 has functional homologies with its mammalian counterpart (human, chimpanzee, pig, bat and mouse), it was clustered together with the other bird species (duck and owl) suggesting an evolutionary divergence for avian species. The third group was composed by a reptile (snake). This avian divergence may suggest an important functional difference (Figure 1B). In mammals, the binding of the IFNs to their receptor initiates a complex signal transduction pathway which results in the activation of IFN regulated/stimulated genes. One of these ISGs, which has been widely studied is *Mx*. In order to confirm whether this was the case in chickens, DF1 wild-type (WT) cells were stimulated with IFNα for 6 h and transcription levels of *Mx* were measured by qRT-PCR (Figure 1C). The results showed that stimulated cells had significantly higher levels of *Mx* transcripts compared to unstimulated cells, indicating a mechanism similar to that found in mammals.

### 3.2. CRISPR Knock out of the ChIfnar1

**Figure 2:**
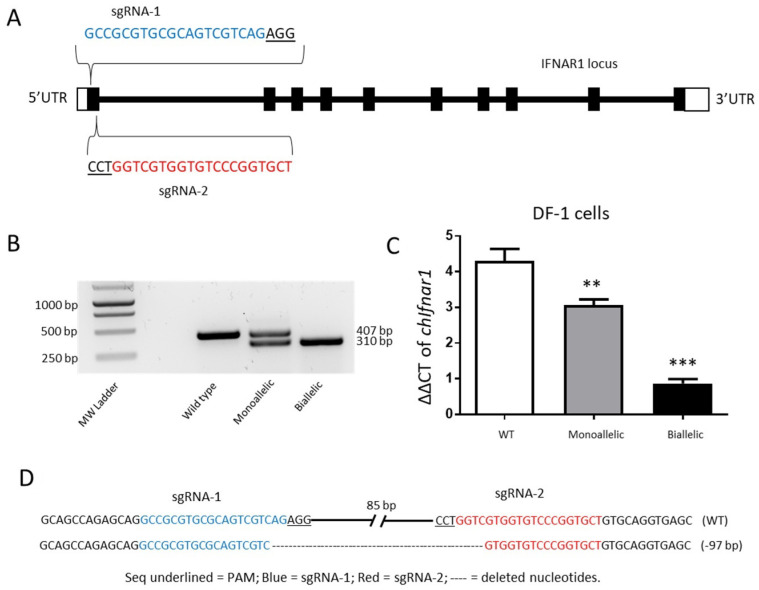
CRISPR/Cas9 mediated deletion of *chIfnar1* Exon1. (**A**) *chIfnar1* structure showing sgRNA targeting. Black boxes = exons, white boxes = 5′ UTR and 3′ UTR. The introns are shown in dark bold horizontal lines connecting the exons. sgRNA sequences and location are shown with PAM sequence is underlined. (**B**) Gel electrophoresis image of Wild type, Monoallelic and Biallelic deletions detected by PCR. (**C**) Expression of *chIfnar1* in WT (white), Monoallelic (grey) and Biallelic (black) assayed by real-time PCR. Data were shown as mean ± SEM (n = 3). Data marked (**) indicates *p* < 0.01, and (***) indicates *p* < 0.001. (**D**) Sanger sequencing of the IFNAR locus confirmed the deletion. Sequences in blue represents the sgRNA-1 and in red the sgRNA-2 sequences, respectively. --- = deleted nucleotides.

In order to study the role of the *chIfnar1* gene in Type I IFN signal transduction we generated a mutant DF1 cell line by editing the genetic sequence of the gene using the CRISPR knock out technique. For this purpose, we designed two sgRNAs, sgRNA-1 and sgRNA-2, both targeting the first exon of the gene (Figure 2A). The transfection of WT DF1 cells generated a deletion of 97 bp in the cellular genome as confirmed by sequencing the genomic DNA of the chIfnar1 locus (Figure 2D). The cells were cloned, and genomic characterization of these clones demonstrated that some of the clones showed a deletion in just one of the alleles, thus generating a monoallelic *chIfnar1^+/−^* clone, whereas other clones showed a deletion in both alleles, generating biallelic *chIfnar1^−/−^* clone. The genomic profile of the clones was further confirmed by PCR analyses and genomic sequencing. It indicated the presence of a double band (WT and deletion bands) for the monoallelic cells, and a single band corresponding to the deletion of a 97 bp fragment in both alleles for the biallelic cells, respectively (Figure 2B). Furthermore, we determined that the deletion also impacted the transcription levels of the gene. The transcription levels for WT, *chIfnar1^+/−^* (monoallelic) and *chIfnar1^−/−^* (biallelic) clones were assessed by qRT-PCR analyses (Figure 2C). As expected, both monoallelic and biallelic chIfnar1 mutants showed a significant reduction of their transcripts compared to the WT DF1 cells. Interestingly, the biallelic clone demonstrated a strong reduction, more than 4-fold compared to WT cells, but not complete ablation of the transcript, indicating that a small amount of transcript containing the deletion is still produced.

### 3.3. Presence and Impact of ChIfnar1 on DF1 Cells

**Figure 3:**
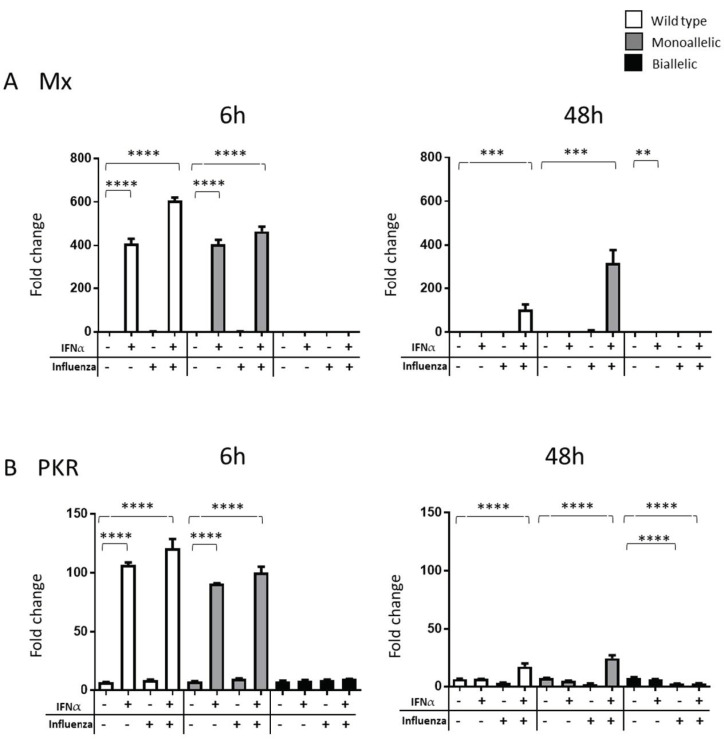
Functional characterisation of *chIfnar1* deletion. After DF1 cells were stimulated with ChIFNα, the cells were harvested at the indicated time points for RNA extraction and cDNA preparation. The transcriptional levels of (**A**) *Mx* and (**B**) *PKR* were measured by real time PCR following IFNα ± influenza infections in WT (white), Monoallelic (grey) and Biallelic (black) cells at 6 h and 48 h post stimulation. Data were shown as mean ± SEM (n = 3). The one-way ANOVA with multicomparisons statistical test was used to compare the differences in relative mRNA levels between ChIFNα ± influenza treatments and DF1 controls at the same time points. A value of *p* < 0.05 was considered statistically significant. Data marked with (**) indicates *p* < 0.01, and (***) indicates *p* < 0.001 and (****) indicates *p* < 0.0001.

Type I IFNs are activated following influenza infection and the role of IFNAR1 in the Type I IFN response to viral infection has been described in detail for mammalian hosts, but such mechanism is still unclear in the chicken. In order to study the impact of the IFNAR1 deletion in the chicken, we investigated the effects of IFNα on the activation of two ISGs, *Mx* and *PKR* [27]. Chicken DF1 WT and chIfnar1^−^/^−^ mutant cells were stimulated with recombinant ChIFNα and infected with Influenza A/ WSN/ 33 (H1N1), either alone or in combination. The transcript levels of *Mx* (Figure 3A) and *PKR* (Figure 3B) were analyzed at 6 and 48 h after ChIFNα stimulation and/or viral infection. Quantitative RT-PCR analyses showed that both *Mx* and *PKR* genes were significantly upregulated in both WT and *chIfnar1^+/−^* monoallelic cells upon stimulation with IFNα alone or IFNα and IAV combined after 6 h of stimulation. Following 48 h stimulation/infection, significant increases in levels of *Mx* and *PKR* expression were detected only when IFNα and influenza were both present, in both WT and monoallelic cells. Results also revealed that the levels of gene transcription for both ISGs are higher at 6 h and tend to decrease at 48 h, suggesting that *Mx* and *PKR* are active in the early stages of infection. While the effect of IFNα stimulation, as suspected, was observed just in the first 6 h, results showed that WSN alone was not able to stimulate the two ISG expressions at either time points. However, *Mx* and *PKR* gene expression was observed at both time points following a combined stimulation with IFNα and WSN. In the case of the 48 h timepoint this indicates a possible synergetic effect of the two agents.

Neither IFNα nor influenza infection were able to stimulate *Mx* or *PKR* in the biallelic *chIfnar1^−^*^/*−*^, which is in agreement with the observation in mammals [28], indicating that activation of *Mx* and *PKR* in chickens is stimulated by Type I IFNs through activation of the Type I IFN receptor IFNAR1. Cells with the IFNAR1 receptor deleted are unable to transduce the IFN signal and activate antiviral effector genes. This would suggest that it is likely that the *chIfnar1^−^*^/*−*^ KO cells had no remaining functional protein.

Interestingly, the basal level *PKR* expression dropped following influenza infection in biallelic *chIfnar1^−^*^/*−*^ knock-out cells.

### 3.4. ChIfnar1 Knock out Impacts the Growth of the Influenza Virus WSN

**Figure 4:**
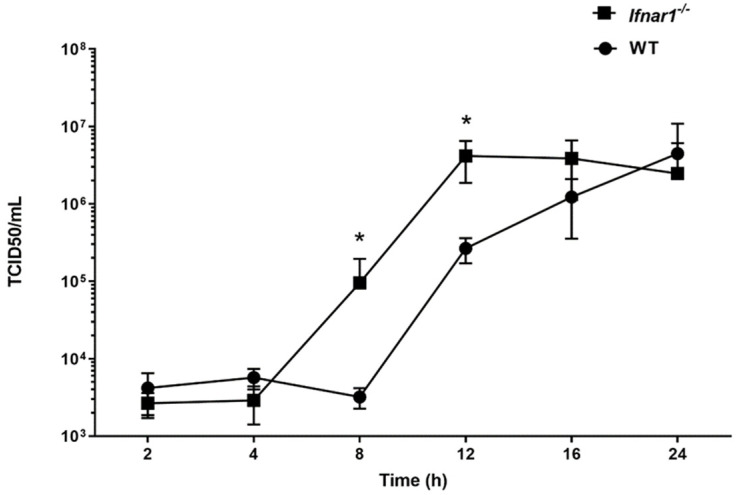
Loss of *Ifnar1* leads to faster viral cycle. DF1 WT (-●-) and *Ifnar1^−/−^* (-■-) cells were infected with Influenza A/WSN/33 (H1N1). At the indicated time post infection, supernatant containing virus was harvested and viral load was measured by TCID50 assay on MDCK cells. Data were shown as mean ± SEM (n = 3). Data marked with (*) indicate *p* < 0.05.

To evaluate the impact of the *chIfnar1* knock-out on the susceptibility to IAV infection, we performed a time course infection experiment on both WT and *chIfnar1^−^*^/*−*^ cells. The WSN influenza strain was chosen as DF1 cells are both susceptible to WSN in the absence of trypsin, and present show a cytopathic effect (CPE). Following infection with Influenza A/WSN at an MOI = 1, supernatant containing virus was collected after 2, 4, 8, 12, 16 and 24 h post infection (hpi) and virus titre was quantified by TCID50 assay (Figure 4). Our data showed that the virus was able to replicate faster as indicated by significantly higher titres in *chIfnar1^−^*^/*−*^ cells compared to WT at 8 h and 12 h post infection (Figure 4). The virus released in the supernatant of the KO cells reached its highest concentration at 18 hpi, compared to 24 hpi in the WT cells (Figure 4), suggesting a shorter virus replication cycle in *chIfnar1^−^*^/*−*^ cells than in WT cells.

**Figure 5:**
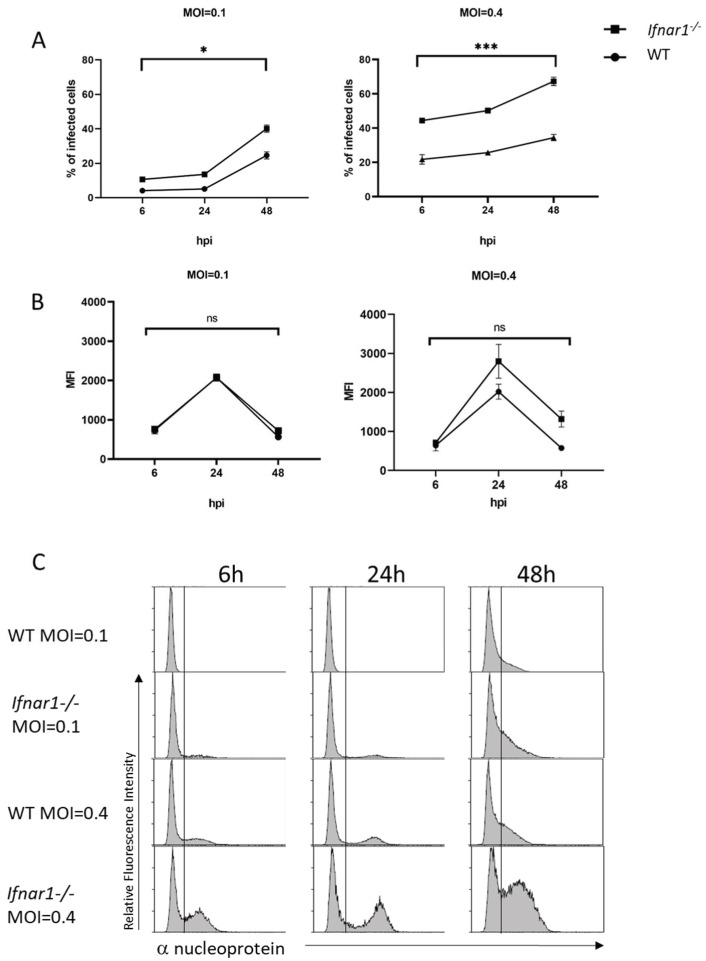
Increases host susceptibility to Influenza virus. (**A**) Percentage of infected cells determined by FACS by measuring the amount of Influenza NP protein at each given time point. DF1 WT (-●-) and *Ifnar1−/−* (-■-) cells were infected with Influenza A/WSN/33 (H1N1) at an MOI = 0.1 and MOI = 0.4 for 6, 24 and 48 h. Data were shown as mean +/− SEM (n = 3). Data marked with (*) indicate *p* < 0.05 and (***) indicates *p* < 0.001. (**B**) Amount of Mean Fluorescent Intensity (MFI) determined by FACS by measuring the amount of Influenza NP protein inside each DF1 cell at each given time point. DF1 WT (-●-) and *Ifnar1−/−* (-■-) cells were infected with Influenza A/WSN/33 (H1N1) at an MOI = 0.1 and MOI = 0.4 for 6, 24 and 48 h. Data were shown as mean ± SEM (n = 3). Data marked with (ns) correspond to non-significant statistical analyses. (**C**) Representative FACS histograms of Influenza infected cells determined by FACS by measuring the Relative Fluorescence Intensity of Influenza NP protein at 6, 24 and 48 h post infection at an MOI = 0.1 and MOI = 0.4.

In addition, to validate the kinetics results, we measured intracellular viral nucleoprotein (NP) by flow cytometry (FACS). WT and *chIfnar1^−/−^* cells were infected with WSN at different MOI (0.1 and 0.4) for 6, 24 and 48 h. Analysis showed higher levels of intracellular viral protein, at 48 hpi compared to the earlier time points (Figure 5A,C) for both cell types. Additionally, a higher percentage of infected cells was observed when cells were infected with a higher MOI = 0.4. However, the percentage of infected cells in the *chIfnar1^−/−^* is higher than in the WT at each time point and different MOIs. These results suggest that *chIfnar1^−/−^* cells are more susceptible to IAV infection than WT.

Mean Fluorescence Intensity (MFI) of both infected WT and mutant cells at each infection time point and infection conditions were then measured to investigate whether the higher susceptibility of *chIfnar1^−/−^* cells is related to the increased presence of viral particles within the cells. No significant difference was observed between WT and *chIfnar1^−/−^* (Figure 5B), suggesting NP content was unaffected.

### 3.5. Role of chIFNAR1 in Influenza Virus Infection

The role of chIFNAR1 in virus infection was further investigated with DF1 WT and *chIFNAR1^-/-^* cells infected with different influenza virus strains, including WSN, Influenza A/Anhui/1/2013 (H7N9) and HPAI Influenza A/Vietnam/1203/2004 (H5N1).

**Figure 6:**
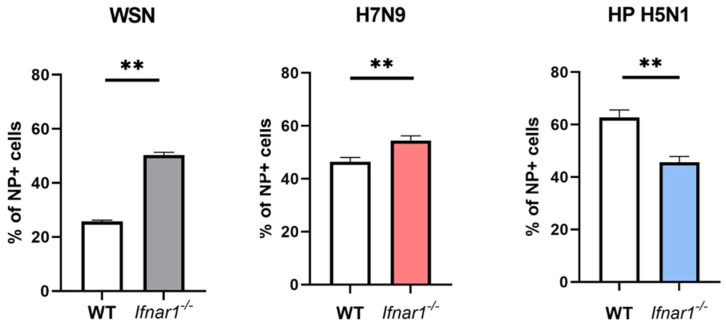
The role of *chIfnar1* in influenza infection with different virus strains. DF1 WT and *Ifnar1−/−* cells were infected with WSN (MOI = 0.4), HP H5N1 (MOI = 1), H7N9 (MOI = 1), and viral infections were measured as percentage of NP positive cells at 24 hpi for WSN and 6 hpi for HP H5N1 and H7N9. All data are representative of one independent experiment in triplicate. Data are shown as mean ± SD and statistical significance was assessed by unpaired two-tailed Student’s *t*-test, ** *p* ≤ 0.01. GraphPad Prism 9 software (GraphPad Software, San Diago, CA, USA) was used for statistical analysis.

At 6 hpi, there was an increased level of NP staining in H7N9 infected *chIFNAR1^−/−^* cells compared to WT cells, a similar observation as found with WSN (Figure 6). In contrast, significant reduction of HPAI H5N1 infection was found in *chIFNAR1^−/−^* cells compared to WT cells (Figure 6). This suggested that different mechanisms were involved in early virus infection when *chIFNAR1^−/−^* cells were infected with high and low pathogenicity virus strains.

## 4. Discussion

Continued circulation of AI viruses in poultry, such as A(H5) and A(H7) viruses, are a public health concern as these viruses cause severe disease in humans. Infections with HPAI virus in gallinaceous poultry leads to rapid onset of severe, systemic disease, often with 100% mortality [29]. The strategies and control measures used to combat HPAI infection including culling and depopulation, cause severe economic losses to the private and public sectors. Therefore, the knowledge of AI virus infection mechanisms and the host immune responses is crucial to understand the viral pathogenesis in birds and to devise novel strategies against these zoonotic agents. While the IFN pathway and its involvement in the host immune response has been well studied in mammalian species including human and mouse, this pathway in chickens remains unclear. A deeper understanding of IFN pathways and antiviral mechanisms may inform novel therapeutic strategies against influenza viruses that pose major health and economic threats. Thus, the aim of our study was to elucidate the role of IFN signaling in chicken, in particular the chIFNAR1 receptor, in the antiviral response against IAV.

As in mammals, the chicken IFNs bind to the IFNAR receptors to initiate an antiviral response. In this study we compared the amino acidic sequences of chIFNAR1 to the human receptor to predict its tertiary structure. Similar to its human counterpart, chIFNAR1 is composed of 3 domains, a cytosolic domain, a transmembrane domain and an ectodomain which is responsible for the interaction with the ligand [30,31]. Comparison between the tertiary structures of the receptors showed structural homology between the two proteins, suggesting a similar function between the receptors. Phylogenetic analyses of the amino acidic sequence from different mammalian, bird and reptile species indicated evolutionarily conserved properties of chIFNAR1. However, the predicted chIFNAR1 receptor topology needs to be confirmed by more accurate methods such as x-ray crystallography.

We confirmed that the IFN signaling pathway is functional in the DF1 cell line as stimulation of WT cells with IFNα was able to induce a significant upregulation of the chicken *Mx* gene, a previously described ISG.

To study the role of IFN response during IAV infection we generated a *chIfnar1^−/−^* DF1 cell line by using CRISPR/Cas9. Molecular characterization of the mutant confirmed the deletion of 97 bp from the first exon of the gene, corresponding to the region targeted by the two sgRNAs. Both the monoallelic and biallelic KO showed a significant reduction of transcription levels. Previous studies have demonstrated, it is critical that both alleles of the gene must permanently be mutated in order to fully inactivate the function of the gene [32]. To further investigate whether the KO of the *chIFNAR1* can influence the IFN signal transduction, we measured the transcription levels of some known ISGs such as *Mx* and *PKR*. Our findings show that the transcription levels of *Mx* and *PKR* are either abolished or strongly reduced in the absence of *chIFNAR1*, suggesting that IFNAR1 is a key component of the Type I IFN signaling. These results are in accordance with what has been previously observed in KO mice lacking IFN receptors, which failed to express Mx1 protein and showed enhanced susceptibility to influenza virus [33]. Thus, the activation of *Mx* and *PKR* in chickens is stimulated by Type I IFNs through activation of the Type I IFN receptor IFNAR1. Cells with an impaired receptor are unable to transduce the IFN signal and activate antiviral effector genes. Intriguingly, our results showed no significant stimulation of *Mx* or *PKR* expression following IAV infection at either 6 or 48 hpi in WT or *chIfnar1^−/−^* cells. The possible explanation is that in vitro virus infection may not induce bioactive IFN and is therefore unable to activate ISG induction. Several studies from different groups also demonstrate that in vitro HP AI virus infection did not induce biologically active Type I IFN in chicken embryo fibroblasts or DF1 cells [34,35]. Viruses may interfere with the IFN response at both the level of IFN synthesis as well as IFN receptor signaling for efficient replication. Indeed, influenza viruses were reported to impair the activation of receptor signaling either by binding viral decoy to Type I IFN to prevent IFN recognition by the receptor, or by degradation of receptors for Type I and Type II IFNs [36], inhibition of JAK/STAT signaling, and regulation of the antiviral function of ISG products [37,38]. Further studies are needed to determine whether influenza virus infection in DF1 cells can induce expression of IFN and a robust IFN response to regulate the antiviral function of ISGs. We were able to demonstrate that a combination of IAV infection and IFNα stimulation were able to induce a strong expression of both ISGs tested. Interestingly, this activation is present still after 48 hpi, whereas either IFNα or IAV alone were not able to induce the expression of the ISGs. This is in accordance with previous studies in chicken showing higher expression levels of ISGs including Mx within a few hours post stimulation [39]. In mammals the antiviral role of Mx following IAV infection has been well characterized [14], studies in chicken have reported controversial results on this type of antiviral response. In vivo studies in chicken either did not demonstrate an antiviral effect against H7N1 [18], or showed just a slight reduction in mortality following H5N2 infection [20]. This lack of protection might be due to the lack of GTPase activity shown by chicken Mx protein [19].

Significantly higher virus titers were observed in *chIfnar1^−/−^* cells at 8 and 12 hpi compared with that of WT cells. This suggests that the anti-viral mechanisms controlled by IFN are limited in the *chIfnar1^−/−^* cells, allowing more viral particles to be released to the cell culture supernatant. A study from Goodman et al., showed increased influenza viral replication in mouse embryonic fibroblasts lacking the IFN receptor [6]. Recently, Carvajal-Yepes, Monica et al. reported enhanced production of a human influenza virus in IFNAR1-knock-down in immortalized chick-derived PBS-12SF cells than parental cells, supporting the importance of the IFN receptor in controlling viral replication [8]. Lack of IFN signaling also has deleterious consequences for virus infection in H7N9 infected DF1 cells, and our study demonstrated increased levels of H7N9 infection in the absence of IFNAR1 compared to WT cells. In contrast, *chIfnar1^−/−^* cells exhibited decreased levels of infection compared to WT cells, when both cell types were infected with a strain of highly pathogenic H5N1 virus. As demonstrated by other studies, no Type I IFN secretion was observed in chicken DF1 fibroblast cells during highly pathogenic H5N1 infection [40]. We hypothesized that while the IFN receptor is necessary to curb viral replication, there would be redundant mechanisms (such as inflammatory response genes) that would be activated as part of the innate immune response upon HP H5N1 infection, even in the absence of a functional IFN receptor. Further experimentation is needed to interrogate the potential mechanisms of the IFNAR1 response to different viral strain infections. However, the subtype-dependent difference observed in our study offers new insights into the possible roles of IFNAR1 in control and modulation of the infection and replication of different strains of influenza A virus.

Furthermore, virus displayed shorter replication cycle in cells devoid of IFNAR1, with detectable virus level in the supernatant from just 8 hpi, in contrast to the detectable virus titers at 12 hpi and onwards in WT cells. Although, the *chIfnar1^−/−^* mutant showed higher viral titres at 8 and 12 hpi, both cell lines reached similar titers at 24 hpi, indicating that there is a maximum (a plateau) number of virions generated within one replication cycle. The higher viral titres observed in *chIfnar1^−/−^* cells could be because of the lack of early innate immune response in these cells, whereas in WT cells the virus needs more time to disrupt the innate immunity defense and subsequently establish virus infection. Thus, in *chIfnar1^−/−^* cells the virus is either able to replicate faster and/or it can infect more cells than in WT. To compliment this theory, we used flow cytometry to measure the amount of viral nucleoprotein synthetized during IAV infection in both WT and mutant cells. Our findings confirm what was observed in the previous experiment, that a higher proportion of *chIfnar1^−/−^* cells become infected as compared to WT. Both these results suggest that the IFN response might impact the entry step of the virus cycle, thus the virus is able to access the cells with impaired IFN response more easily than the cells with a normal IFN pathway. Our results indicate that neutralizing the IFN response in cell lines can improve the production of influenza virus and offer a viable alternative system for the production of influenza virus vaccines.

Our overall findings demonstrate that IFNAR1 in chicken is a key component of the IFN transduction signal pathway. It plays a similar role as its human homolog, in its absence, IFN are not able to stimulate the expression of ISGs. Cells with an impaired IFN receptor are more susceptible to the influenza virus infection. Moreover, the lack of chIFNAR1 impacts the entry process of the virus confirming that the innate immunity is a crucial component of the host antiviral defense. We used chicken fibroblast cell line DF1, a homogeneous cell population, for these experiments since they allowed us to study the signaling pathways without immune cell infiltration, which can confound results observed for an animal model. However, it should be stated that by infecting macrophages, dendritic cells, or lung epithelial cells isolated from chickens lacking interferon receptors will enable us to better understand immunity during influenza virus infection. Our findings bring new contributions to the field of host-avian pathogen interaction and innate immunity in chickens. The outcome of our study might have huge implications in the manufacturing of influenza vaccines, as embryonated chicken eggs are the main bioreactors of the seasonal influenza vaccine.

## 5. Conclusions

These studies represent an important finding with regard to the chicken host response to AI virus. We have demonstrated that the role of IFN signaling in response to infection is diverse and can lead to both pro-viral and anti-viral effects. We were able to demonstrate that in the absence of IFNAR1, cells were not only more susceptible to the influenza virus, but also that there was a reduction in replication cycle. Whilst IFNAR1 behaved similarly to mammalian IFNAR1, in that it was able to trigger ISG and reduced viral replication, in the case of HP H5N1 the converse was observed, and viral replication increased. Further investigation into the mechanisms that lead to increased viral replication may provide valuable insights into both the biology of HPAI virus as well as possible means of intervention.

## Figures and Tables

**Table 1:** PCR and qRT-PCR primers and probes sequences.

Target Gene	Primer/Probe	Sequence (5′–3′)	Accession No.
GAPDH	F	CCCCAATGTCTCTGTTGTTGAC	AF047874
	R	CAGCCTTCACTACCCTCTTGAT	
	Probe	CTTGGCTGGTTTCTCC	
IFNα	F	GGACATGGCTCCCACACTAC	X92476
	R	TCCAGGATGGTGTCGTTGAAG	
	Probe	CAGCGCGTCTTGCTC	
PKR	F	GCAGAAGTAAGAGTGAGGCAAATGA	HQO14737
	R	GCCACCTTTACCAATAGGCTCTAT	
	Probe	CTGTGGATGAAAGGTTTC	
Mx	F	GTCCAAGAGGCTGAATAACAGAGAA	CR 389077
	R	GGTCGGATCTTTCTGTCATATTGGT	
	Probe	CTGCTGCCTCATCCTT	
IFNAR1	F	CGGCCACCCAAACCTTAGAA	Gene ID: 395665
	R	CCATCTCGCAGCAGTTGTCT	
